# Wearable Smart Gloves for Optimization Analysis of Disassembly and Assembly of Mechatronic Machines

**DOI:** 10.3390/s25175223

**Published:** 2025-08-22

**Authors:** Chin-Shan Chen, Hung Wei Chang, Bo-Chen Jiang

**Affiliations:** Department of Mechanical Engineering, National Pingtung University of Science and Technology, Pingtung 912301, Taiwan; a0988908001@gmail.com (H.W.C.); destiyff@gmail.com (B.-C.J.)

**Keywords:** disassembly and assembly, smart gloves, performance measure functional, optimal path, optimal work table height, inertial measurement unit, thin-film force sensor

## Abstract

With the rapid development of smart manufacturing, the optimization of real-time monitoring in operating procedures has become a crucial issue in modern industry. Traditional disassembly and assembly (D/A) work, relying on human experience and visual inspection, lacks immediacy and a quantitative basis, further affecting operating quality and efficiency. This study aims to develop a thin-film force sensor and an inertial measurement unit (IMU)-integrated wearable device for monitoring and analyzing operators’ behavioral characteristics during D/A tasks. First, by having operators wear self-made smart gloves and 17 IMU sensors, the work tables with three different heights are equipped with a mechatronics machine for the D/A experiment. Common D/A motions are designed into the experiment. Several subjects are invited to execute the standardized operating procedure, with upper limbs used to collect data on operators’ hand gestures and movements. Then, the measured data are applied to verify the performance measure functional best path of machine D/A. The results reveal that the system could effectively identify various D/A motions as well as observe operators’ force difference and motion mode, which, through the theory of performance indicator optimization and the verification of data analysis, could provide a reference for the best path planning, D/A sequence, and work table height design in the machine D/A process. The optimal workbench height for a standing operator is 5 to 10 cm above their elbow height. Performing assembly and disassembly tasks at this optimal height can help the operator save between 14.3933% and 35.2579% of physical effort. Such outcomes could aid in D/A behavior monitoring in industry, worker training, and operational optimization, as well as expand the application to instant feedback design for automation and smartization in a smart factory.

## 1. Introduction

Along with the development of industrial technology, traditional manufacturing needs to integrate modern technologies, such as the Internet of Things, big data, and artificial intelligence, to achieve intelligent, automated, flexible, and digitized production [1,2]. Real-time monitoring and control in the production process require the support of precise, reliable, and instantaneous sensing technology to enhance operational visibility, improve work efficiency and quality, and reduce defective products and labor costs.

Automated production systems emerge to meet the market demand for small quantities and a diverse range of products; nevertheless, assembly work remains one of the most important processes in molding instruments or equipment. Assembly work typically accounts for approximately 60% of the overall production time for equipment. Disassembly and assembly work often involve human operation, which directly affects quality and security due to the applied force and precision of motion. However, traditional monitoring methods (video surveillance, personnel checks) cannot immediately and accurately record detailed operating behaviors.

Wearable sensing devices, with their advantages of simplicity, handiness, high sensitivity, precision, and flexibility, are being introduced to disassembly and assembly work for providing continuous, non-invasive measurements of motion and subtle force information, presenting the potential for big data analysis of operating behaviors and fatigue detection.

Wearable sensing devices are broadly applied to sports, industry, healthcare, and environmental monitoring. Past research has revealed that wearable devices are used to track the number of steps and heartbeat, while inertial measurement units are utilized for interpreting gestures and measuring gaits; however, they are seldom applied to research on the disassembly and assembly work of equipment.

This study, by installing the thin-film force sensor and IMU-integrated wearable devices on factory workers’ mechatronics machine disassembly/assembly work, aims to discuss the response of distinct motions (tightening, loosening, crimping, drawing, moving) in the disassembly/assembly process to sensing data as well as realize operators’ motion behaviors of posture, finger force, and pressure in the real-time monitoring mechatronics machine disassembly/assembly process. The best path, time, and work table height in the equipment disassembly and assembly process can be determined through the analysis of force and moment of inertia. Moreover, it is expected to provide a valuable reference for human–robot collaboration in disassembly and assembly procedures for robotic arms, as well as for smart manufacturing disassembly and assembly work, intelligent factories, and logistics systems.

## 2. Related Works

A significant discussion of mechatronics follows. Mechatronics, which combines mechanical engineering, electronics, control theory, and information technology, is at the core of modern smart manufacturing and automation systems. Along with the rapid development of artificial intelligence and the Internet of Things, the application of mechatronics is expanding, covering various levels from intelligent robots to automated production lines. For instance, Lettera et al. [3] and Park et al. [4] discussed the integration of AI and electromechanical systems to enhance system intelligence. Cintra Faria et al. [5] deeply analyzed the role of mechatronics in innovative product development. Such research emphasized the critical status of mechatronics in modern engineering and indicated that future research should focus on deepening interdisciplinary integration and intelligent applications.

A significant discussion of equipment D/A work follows. The disassembly and assembly work of equipment play a key role in product lifecycle management, particularly at the stages of maintenance, upgrade, and recycling. Along with the advancement of automation technology, the promotion of efficiency and security in disassembly and assembly work has become a research focus. For example, Mohammed Eesa et al. [6] discussed the application of robot teardown to handle obsolete products, particularly the challenges and solutions for task and exercise planning. The disassembly/assembly work analysis and the overview of modeling technology provided by Iacob et al. [7] offer guidance on promoting disassembly/assembly work simulation and planning. Such research indicated that future disassembly and assembly work should be developed towards higher automation, intelligentization, and flexibility to cope with diverse product designs and rapidly changing market demands.

The application of thin-film sensor technology to D/A work is as follows. Thin-film sensors, due to their characteristics of being lightweight, flexible, and highly sensitive, are widely applied to various sensing scenarios, particularly in disassembly and assembly work, which require precise monitoring. For instance, Schmaljohann et al. [8] developed thin-film sensors suitable for both plane and curved surfaces, demonstrating the potential for tool wear monitoring and force sensing. Song et al. [9] proposed a fast interface self-assembly technique to prepare a uniform nanoparticle monolayer thin film, which provided a new method for flexible electronics and sensor manufacturing. Such research revealed the broad potential of thin-film sensor technology for applications in disassembly and assembly work. The performance and reliability under distinct operating environments should be further explored.

The application of inertia sensors to D/A work is as follows. Inertia sensors, which provide real-time data on object movement, present significant benefits in dynamic monitoring and control during disassembly and assembly work. Zheng et al. [10] discussed the modeling and compensation of inertia sensor errors, presenting the guidance value to enhance sensing precision and system stability. García-de-Villa et al. [11] reviewed the application of inertia sensors to human movement analysis and provided a comprehensive perspective for sensor selection, data processing, and application scenarios. Such research indicated that applying inertia sensor technology to disassembly and assembly work could enable precise monitoring and intelligent control during the operating process. The application strategies and integration methods under a complicated environment should be further studied.

A discussion on the Theory of Robotic Arm Path Planning follows. In modern automation and intelligent manufacturing systems, robotic manipulators are extensively employed for tasks such as material handling, assembly, and precision machining. To ensure operational safety and efficiency, path planning represents a critical technological component. Its primary objective is to determine an optimized trajectory that adheres to multiple kinematic and environmental constraints. Commonly utilized methodologies include the Penalty Function Method—an established and effective technique for constrained optimization—and the performance measure functional approach, which is particularly prevalent in dynamic systems, optimal control, and trajectory optimization. These methods may be applied independently or in combination to design safe and efficient motion paths. Fundamentally, this constitutes a multivariable optimization problem subject to complex nonlinear constraints, often addressed using the calculus of variations, Euler–Lagrange equations, or numerical optimization techniques.

An overview of the performance measure functional is provided as follows. Performance measure functional (PMF), one of the common tools to solve optimization problems, is frequently applied to evaluate functions requiring the definition of “overall system performance”, such as the integration of a complete trajectory, process, or output, or the sum of performance indices. Such a method is commonly used in dynamic systems and path planning, particularly in optimal control and trajectory optimization.

The fields of smart manufacturing, medical support, and service robots, including robotic arms with high precision and flexibility, are widely applied to various complex tasks, such as precision assembly, welding, and autonomous carrying. To enhance the task performance of robotic arms under dynamic and unstructured environments, it becomes a core challenge to plan a secure and performance-efficient movement path [12].

Traditional robotic arm path planning emphasizes geometric feasibility, such as obstacle avoidance and joint limitation, but seldom systematically considers overall movement performance, including energy consumption, time, smoothness, and dynamic stability. To solve such problems, a PMF is introduced as the reference for evaluation and optimization. The core idea is to quantify the “overall behavior” of robotic arms by integrating certain physical or geometric properties of the entire path to create an object that can be optimized [13]. Garriz et al. [14] considered both minimal energy consumption and performance maximization when discussing the optimization of robotic arm trajectories, presenting a comprehensive PMF design and experimental verification. Jeong et al. [15] proposed optimizing sensing and control performance in the fields of smart control and path planning, providing valuable reference values for enhancing the movement positioning of autonomous robots. Manrique Córdova et al. [16] discussed the complex path planning of mobile robots to present a reference value for the design of PMF functions and the integration with metaheuristics.

Chen [17] combined PMF and physics constraints conditions (e.g., collision avoidance, joint limitation) in the research to significantly enhance the feasibility and quality of the robotic arm path. Additionally, the shortest path, the safety indicator of the path being used, and the time required for the PMF were considered to discuss and plan dual robotic arm obstacle avoidance technology and optimized paths, as well as the physical strength required by operators to carry workpieces during the disassembly and assembly processes. Accordingly, the PMF indices in this study are planned as minimal time, secure path, shortest path, minimal energy consumption, and vibration avoidance, where the minimal energy consumption functional could enhance artificial disassembly/assembly or the battery life efficiency of robotic arm operation. The minimal acceleration functional effectively reduces movement vibration and energy consumption. The PMF of the machine disassembly and assembly in this study can be described in Equation (1).

(1) $$J\left(P\right)=D\left(P\right)+ℷ\;S\left(P\right)+W\left(P\right)\;\;\lambda \geq 0,$$

where D(**P**) is the length value of the path, S(**P**) is the value of security, W(P) is the value of energy, and λ is the weighting factor.

The length value of the path can be defined using the arc length of the path, i.e., Equation (2), as follows:

(2) $$\begin{array}{c} D\left(P\right)=\int_{t_{0}}^{t_{f}}\|\dot{X}\;\left(t\right)\|dt\\ =\int_{t_{0}}^{t_{f}}\sqrt{{\dot{X}}_{1}^{\;\;2}\left(t\right)+{\dot{X}}_{2}^{\;\;2}\left(t\right)+{\dot{X}}_{3}^{\;\;2}\left(t\right)}\;dt, \end{array}$$

The value of the path security can be defined using the arc length of the path, i.e., Equation (3), as follows:

(3) $$S\left(P\right)=\int_{t_{0}}^{t_{f}}\|X\left(t\right)-Ob\left(t\right)-\varepsilon \;\|dt,$$

The value of the path energy can be defined using the arc length of the path, i.e., Equation (4), as follows:

(4) $$\begin{array}{c} W\left(P\right)=\int_{x_{0}}^{x_{f}}F\;•\;dX\\ =\int_{t_{0}}^{t_{f}}F\;•\;\dot{X}\;\left(t\right)dt, \end{array}$$

where ‖▪‖ represents the norm, ***X***(*t*) is the wrist position, ***Ob***(*t*) is the obstacle position, and ε is the safety clearance.

A path planning theory framework with a PMF as the core and the complete D/A procedure evaluation is proposed in this study. Wearable devices integrating thin-film force sensors and IMUs are installed on factory workers for the practical verification of mechatronics machine disassembly and assembly work. They present good practicability and promotional potential for applications to multi-degree-of-freedom industrial robotic arms, collaborative robots, and high-level humanoid arms.

## 3. Materials and Methods

This chapter introduces the hardware architecture, software architecture, experimental design process, and method of this study.

### 3.1. Disassembly and Assembly System Development

**Figure 1:**
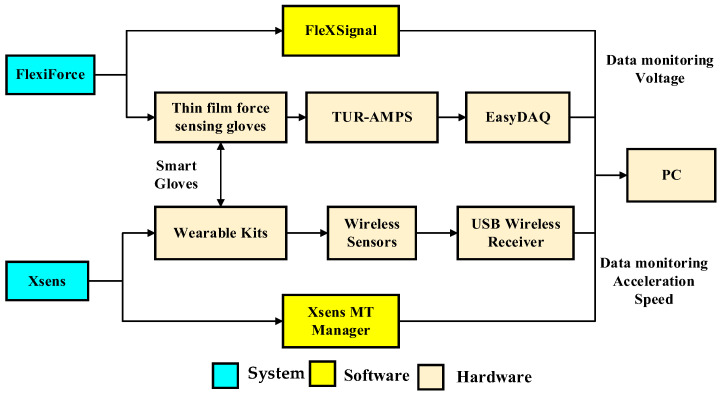
FlexiForce system and Xsens system architecture diagram.

The system architecture for this study is divided into three parts: the Xsens system, the FlexiForce system, and the material sorting and processing machine. The Xsens system and the FlexiForce system include relevant sensor kits and corresponding monitoring programs for monitoring acceleration, speed, and voltage data (Figure 1). The material sorting and processing machine is a mechatronics device that is disassembled and reassembled during the experiment, comprising D/A modules as well as a piping and wiring module.

#### 3.1.1. Xsens System

Xsens MVN (Movella Holdings Inc., El Segundo, CA, USA) [18], a motion capture system developed by Xsens in the USA, can real-time record and capture 6-degree-of-freedom data on the human body and skeleton using micro-inertial sensors. The Awinda series of Xsens MVN is utilized in the experiment presented in this study. The Awinda series, a wireless transmission version with built-in magnetic field compensation technology, can significantly reduce the calibration time for each experiment. By adding sensors, it could expand the capture part and range to more accurately and stably track body movement data.

The Awinda Starter series, which contains 17 wireless sensors (version 2.0.0), is fixed to the body through the wearable kit and transmits and receives data via a USB Dongle antenna. Each sensor contains a set of 3-axis accelerometers and 3-axis gyroscopes. The output monitoring software, Xsens MT Manager (version 2020.5.0), could output six categories of data, covering inertial data, location data, direction data, environmental data, time data, and coordinate data. The data captured in this study are mainly inertial data. During the experiment, the sampling rate of the Xsens MT Manager was set to 2 kHz.

#### 3.1.2. FlexiForce System

FlexiForce, a single-point thin-film force sensor developed by Tekscan Inc., Norwood, MA, USA, is primarily used for measuring and monitoring changes in an object’s pressure, weight, and force. A thin film, the key part of a thin-film sensor, contains piezoelectric material. When thin-film sensors receive external force (pressure), the material deformation presents a good linear relationship with the external force. Thin-film force sensors, Model No. FlexiForce A201 [19], matched with 8 electronic amplifiers (TUR-AMP) to provide 0–5 V analog voltage output, are used in this study. They are connected to the TUR-AMP end via the LEMO interface of the EasyDAQ data acquisition system and further connected to the computer end through a USB plug, allowing for multiple data collection and real-time visual data monitoring with FlexSignal applications. The sampling rate of the EasyDAQ system (MEMS Technology Corp., New Taipei City, Taiwan) was 48 kHz, with an analog-to-digital resolution of 14 bits, which is sufficient for capturing and analyzing data during the disassembly and assembly processes.

The integration and development of wearable devices with various sensors have grown rapidly and are widely applied across different fields. For example, Tamantini et al. [20] developed a wearable smart garment—an instrumented intelligent shirt—for workplace risk prevention, aiming to enhance safety and well-being in occupational environments. Additionally, Di Rienzo et al. [21] proposed an industrial safety sensing glove based on Near-Field Communication (NFC) technology to monitor workers’ operational behavior and proactively report hazardous situations, thereby preventing accidents and injuries.

**Figure 2:**
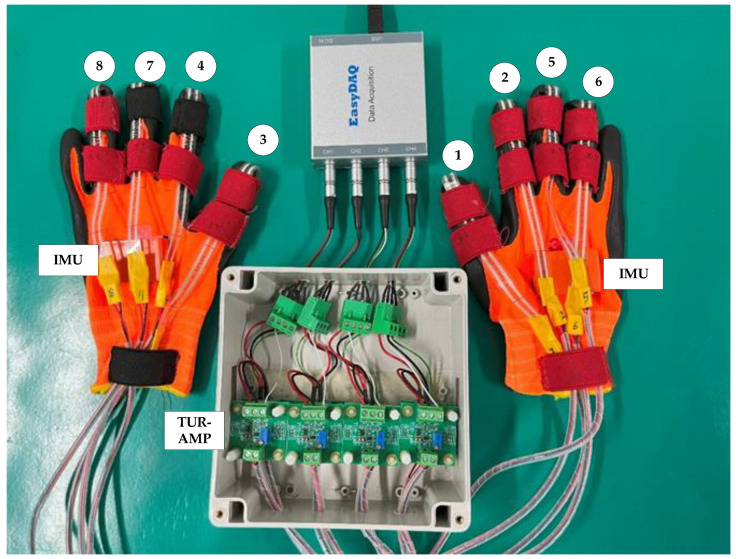
Smart gloves and sensor capture channel mapping diagram.

For connecting the self-made smart gloves with sensor channels, comfort grip gloves (sized XXL, 20 cm) from 3 M, USA, are selected for this study. First, the stretch fabric is sewn to the parts of the wrist, the distal knuckles of the fingers, and the proximal knuckles of the glove. A hook and loop fastener is sewn on the back of the hand in order to concentrate various channel wires and avoid wire loss caused by frequent movement during disassembly/assembly. Stretch fabric is sewn to the proximal knuckles to secure the thin-film sensors on the fingers. Sewing stretch fabric on the parts of the distal knuckles could protect thin-film sensors. The use of little finger in the disassembly/assembly process is relatively low, and the data acquisition system could simultaneously monitor 8 channels; therefore, thin-film sensors are merely used for thumbs (Ch01, Ch03), index fingers (Ch02, Ch04), middle fingers (Ch05, Ch07), and ring fingers (Ch06, Ch08) of both hands to monitor force. The integrated glove is shown in Figure 2. The channels are allocated as follows: Ch01, Ch02, Ch05, and Ch06 for measuring the right hand, and Ch03, Ch04, Ch07, and Ch08 for measuring the left hand, to avoid wire knotting during the work.

#### 3.1.3. Mechatronics System

**Figure 3:**
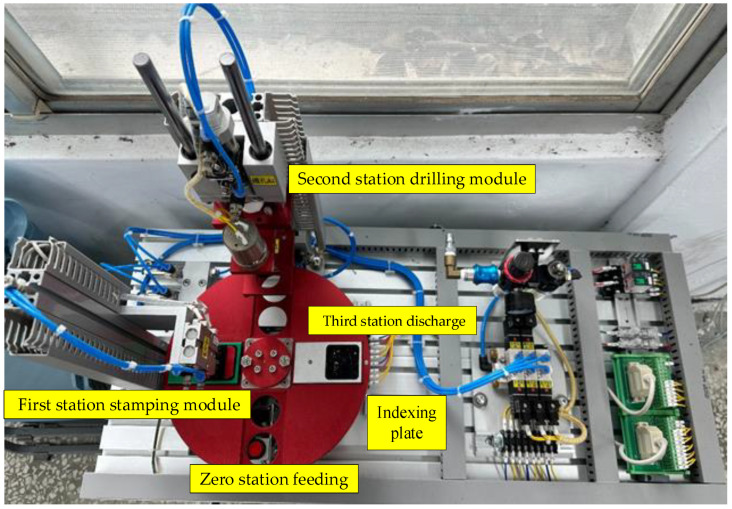
Material sorting and processing machine structure physical diagram (FETON Automation Industrial Co., Ltd., Kaohsiung, Taiwan).

The disassembled and assembled machine for this study is a material sorting and processing machine, as shown in Figure 3. According to the working sequence, the station is classified into four modules: zero station feeding, first station stamping module, second station drilling module, and third station discharge. The actual locations are related to the module design. The basic operation process of the machine is described as follows. A workpiece is fed from the zero station, where the material sensor identifies the workpiece as either metal or non-metal material, and it is then rotated through a dividing table to the change station. When the workpiece is a non-metal material, the dividing table rotates the workpiece to the first station for stamping and then to the third station for discharge. When the workpiece is a metal material, the dividing table first rotates the workpiece to the first station for stamping and then to the second station, where it is fixed with a gripper for drilling, and finally to the third station for discharge. The equipment control functions cover emergency stop, restoration, single cycle, continuous cycle, and jump cycle.

The upper layer of the material sorting and processing machine is the operation layer of the mechanism, with major mechanism modules of drilling cylinder module (about 3.9 Kgw), stamping cylinder module (about 1.3 Kgw), dividing table module (about 6.3 Kgw), inductive anti-reverse valve (about 0.4 Kgw), relay module, terminal block module, solenoid valve assembly, and air regulator. The lower layer is composed of the electromechanical control operation panel of the machine and a Mitsubishi programmable logic controller (FX3U-32MR).

A personal computer (ASUSTeK Computer Inc., Taipei, Taiwan, CPU Intel i7-8700, GPU NVIDIA GeForce GTX 1060 6 GB, memory 8 GB DDR4, hard drive 1.5 TB, WIN 10 operating system) is used in this study for the real-time data monitoring and recording of Xsens MT Manager and FlexSignal during disassembly/assembly, as well as exporting data to Excel for data analysis.

### 3.2. Experimental Design

To ensure the consistency, efficiency, security, and quality in the product production process, dismantling/assembly planning is generally applied to disassemble/assemble mechanism parts in modern industry in order to find out the best program for disassembling/assembling different parts into final products, reduce the complexity in disassembly/assembly procedure, and find out the best product assembly for reducing costs and enhancing efficiency, quality, and security.

Considering that, after procedure evaluation criteria, a suitable disassembly/assembly procedure could be planned according to various factors and preceded actual disassembly/assembly work, a complete AP procedure evaluation, as shown in Figure 2, could be broken down into dismantling/assembly planning (D/ASP), dismantling/assembly line balance (D/ALB), and dismantling/assembly path planning (D/APP) [22].

The basic design of the experiment is explained below.

- Operators in the experiment: Three male and one female operators, with heights ranging from 153 to 193 cm, participated in the research, following the standard disassembly and assembly process.
- Disassembly and assembly of objects in the experiment: All mechanism modules on the upper layer of the machine are separated from the substrate and then assembled on the machine board, and their normal function is tested.
- Disassembly/assembly machine and work table design in the experiment: The machine height, in the experiment process, is fixed at 100 cm, and the height-adjustable work table is then placed and set at heights of 80 cm, 100 cm, and 120 cm to discuss the work efficiency of the dominant hand and the non-dominant hand. The work tables are positioned on the left and right sides of the subjects for the disassembly and assembly work experiment.
- Tools for disassembly/assembly process in the experiment: Material sorting and processing machine fastening screws for wiring are classified into M2 slotted flat-head screws for terminal block wiring fastening and M3 cross-recessed pan head screws (with washers) for the rest; regarding screws for fastening four major modules on the machine base, 2 M6 hex socket screws are used for the inductive anti-reverse valve and 2 M8 hex socket screws are used for the rest modules. The used tools include a blue slotted screwdriver (2.5 mm × 0.5 mm × 75 mm), a red Phillips screwdriver (PH0 × 75 mm), a black No. 5 (M6) hex key, and a black No. 6 (M8) hex key. Machine screw disassembly and assembly are uniformly spun 5 times for fastening or loosening.
- Disassembly/assembly process design for this experiment: All mechanism modules should be disassembled from the substrate in the disassembly process. According to the AP evaluation points, the major mechanism disassembly process involves disassembling the pneumatic piping, secondary wiring, pneumatic inductive anti-reverse valve, drilling cylinder module, stamping cylinder module, and dividing table module. The assembly sequence is reversed. The experiment is completed after assembly and function testing.

Moreover, factory workers, during disassembly and assembly work, stand stably on their lower limbs without large gait movements. They stand on fixed areas to complete all operations, without having to carry modules over long distances. In this case, the lower limbs are merely adjusted to achieve a standing posture and balance. Furthermore, the motion focuses on the arms and trunk, specifically between the waist and shoulders, during the module operation process. Additionally, the operated module weighs less than 6 kg so that factory workers are not required to exert force with their lower limbs. As a result, the data analysis extracts the data from the inertia sensors and thin-film sensors on the self-made smart gloves during the disassembly and assembly processes.

### 3.3. Experimental Process

The sizes of the machine and the adjustable table are first measured to position the mechatronics machine. The work table is then lifted or descended to the experimental height and placed at a distance of an arm’s length away from the operator.

**Figure 4:**
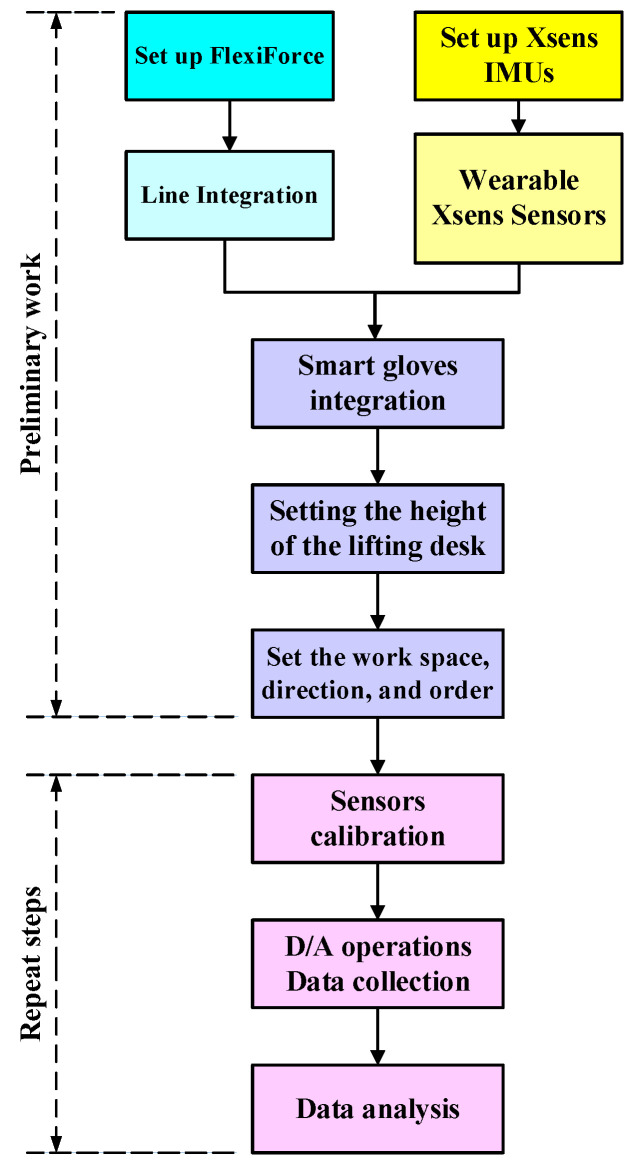
The main process of the disassembly and assembly experiment.

**Figure 5:**
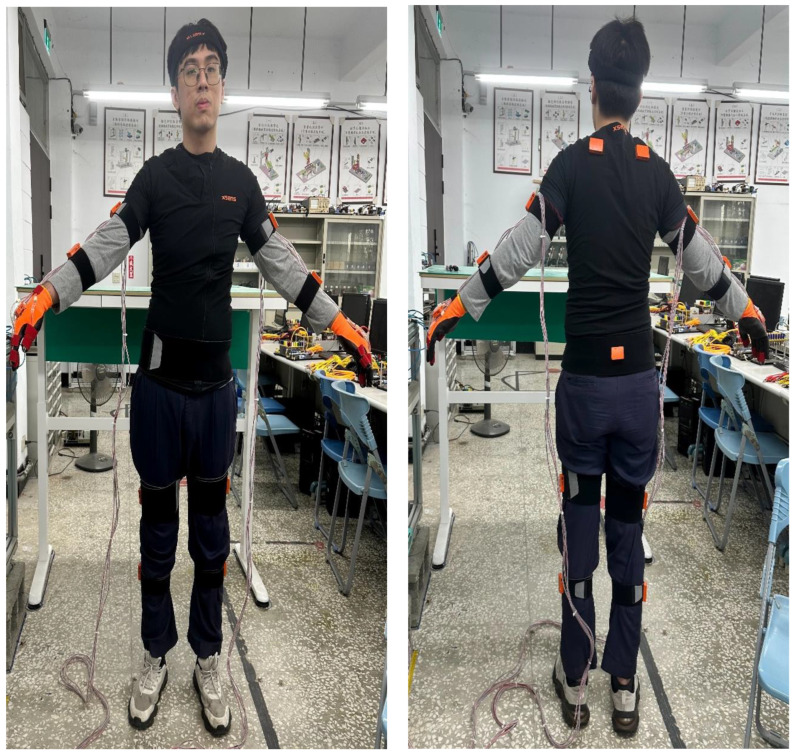
The operator is wearing Xsens sensors (Movella Holdings Inc., El Segundo, CA, USA) and smart gloves.

Figure 4 is the flowchart of the experiment in this study. First, FlexiForce thin-film sensor gloves and Xsens inertial measurement wireless sensors are integrated. Disassembly/assembly machine, work tables, and disassembly/assembly tools are then positioned. The equipment, circuits, and space required for the experimental process are defined and integrated to minimize interference during the disassembly and assembly processes. Xsens sensors are connected with wireless transmission. Although circuit integration is not necessary, the location of Xsens sensors should be predetermined, and the installation location and gap should be defined to minimize experimental differences. The smart thin-film sensor gloves in this study are equipped with eight FlexiForce thin-film sensors to measure the subtle forces of the fingers during disassembly and assembly. Meanwhile, the back of both hands is equipped with fixed Xsens sensors to measure dynamic inertial information during disassembly and assembly (Figure 5). The work table height and workspace are then adjusted for the disassembly and assembly experiment. The disassembly and assembly sequence is planned to confirm the machine height, the height of the adjustable table for placing parts, the disassembly and assembly sequence, and the operation space between the machine and the table. All FlexiForce thin-film sensors and Xsens sensors are calibrated before the experiment, and communication transmission is confirmed to be normal before recording experimental data. The disassembly mechanism module is planned according to AP. The mechanism module is assembled after the disassembly work is completed. Functions are tested after completing assembly. Parameter selection and output are performed after the experiment is completed for final force data analysis and inertia data analysis.

## 4. Results and Discussion

Aiming at a path planning experiment applying an inertial measurement unit to the mechatronics equipment disassembly/assembly, the research results are explained as follows.

### 4.1. Xsens Inertia Experimental Result

In the experiment, the mechanism and piping of the mechatronics are disassembled and placed on adjustable work tables with three different heights. The mechanism and piping assembly are then installed, and the PMF is used to operate the collected data to acquire the best data. Two types of screws should be spun five times in the assembly/disassembly process to tighten the mechanism. A more precise measurement of the losing/fastening force is obtained by analyzing data from smart gloves (explained in Section 4.2).

**Figure 6:**
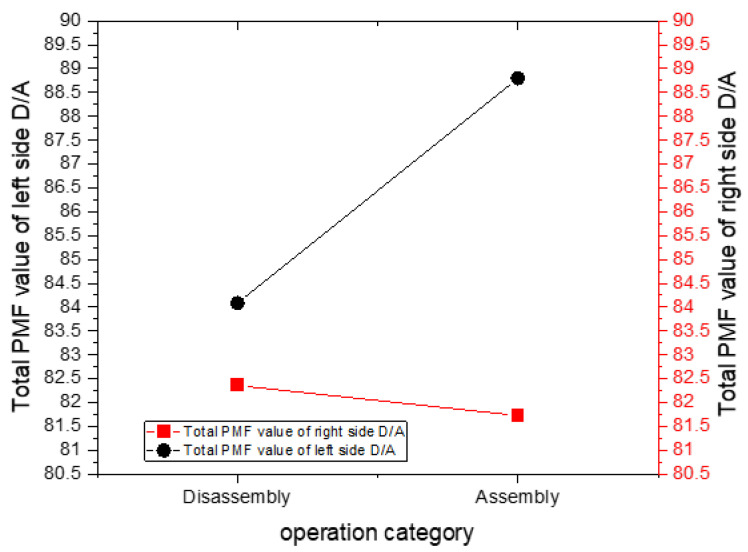
The total performance index function value during disassembly and assembly.

Figure 6 shows the work table being placed on the left or right side of the operator to sum up the performance index function value during disassembly. It reveals that the performance index function sum is small when executing disassembly with the work table on the right side. Accordingly, the dominant hands of the three factory workers are their right hands, and the tolerance side is the right hand when disassembling on either the left or the right. In this case, placing work tables on the right side of factory workers, with the dominant hand being the right hand, is the better location.

Figure 6 illustrates the cumulative value of the performance index function during the assembly process. Three factory workers with the right hand as the dominant hand present the tolerance side as the dominant hand when preceding left-sided assembly work. The right-side performance index function value is, therefore, smaller, with the sum being 81.726. When preceding assembly work on the left side, the tolerance side, in the assembly process, is performed with the non-dominant hand, for which the performance index function value is larger, with a sum of 88.80328. The average error here is 0.530102, while the average error of the assembly is 1.179553.

**Figure 7:**
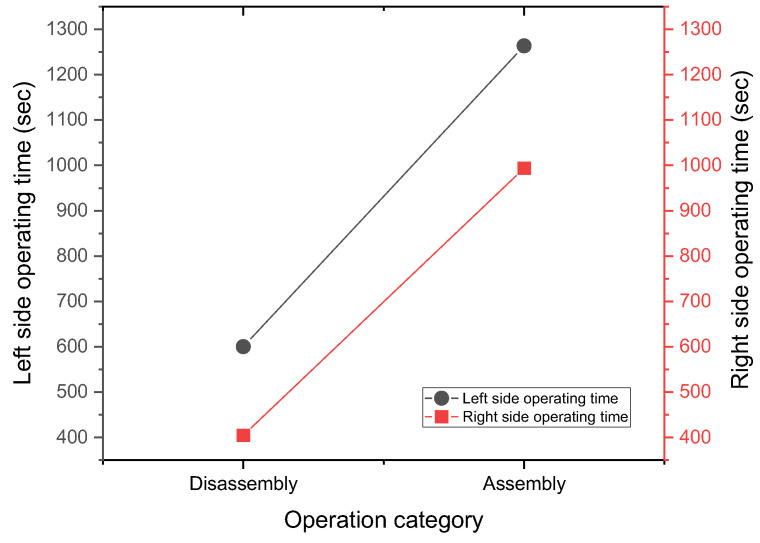
The total operating times of three factory workers during disassembly and assembly.

The operating times of three factory workers are shown in Figure 7. The sum of the disassembly and assembly times on the right side and the left side appears as 404 s and 993 s, respectively. The operating time for the right side is less than that of placing the adjustable work table on the right side, which is the best operating direction.

The average error time for the disassembly mechanism is about 43.67 s (the maximum difference is 84.00 s), and the average error time for the assembly mechanism is about 51.56 s (the maximum difference is 67.44 s).

**Figure 8:**
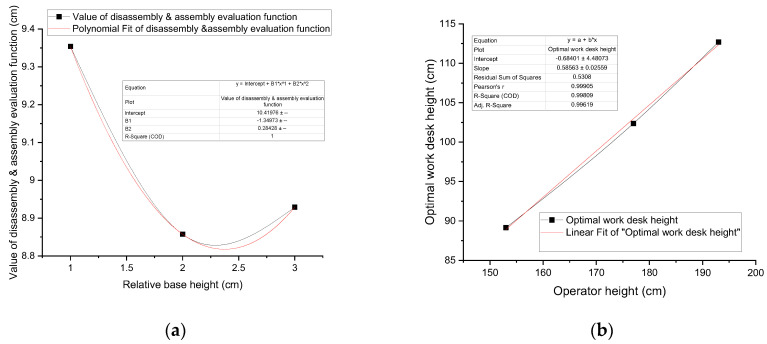
Optimal work table height for workers of different heights: (**a**) the average D/A performance index function value of the operator at a height of 153 cm; (**b**) the linear fit of optimal work desk height for different workers’ heights.

Figure 8a shows the average disassembling and assembling performance index function value of the operator at a height of 153 cm under different-height work tables. The regression analysis yields an optimal value of 8.828094574, indicating that the best work table height for a 153 cm factory worker is 89.15309457 cm.

Similarly, the optimal disassembly/assembly performance index function value for the 177 cm factory worker is 8.570790485, corresponding to the optimal work table height of 102.3807905 cm. Moreover, the optimal work table height for a 193 cm factory worker is 112.696309 cm.

For factory workers with varying heights, linear regression can be used to determine the optimal work table height that aligns with the best ergonomic design curve. As shown in Figure 8b, the linear equation is y = 0.5856X − 0.684, where X is a factory worker’s height and y is the best work table height corresponding to the factory worker standing for operation. Workstations require personnel rotation, which can assign work with adjustable height and quickly calculate a suitable value using the above equation for the work table design curve. This allows for rapid adjustment of the work table height, making the operation comfortable and smooth. The design adheres to another work table design principle, namely, adding 5–10 cm to the elbow height (the elbow heights of the three operators being 94.095 cm, 109.917 cm, and 120.625 cm).

**Figure 9:**
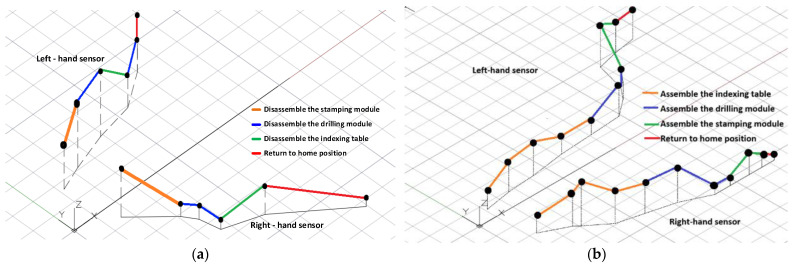
The shortest disassembly/assembly path planning diagram: (**a**) the shortest disassembly path planning diagram; (**b**) the shortest assembly path planning diagram.

The experiment reveals that when a work table is placed on the right side with the same height as the mechatronics machine, the average performance index function value is the lowest, and the operating time is the shortest. The shortest disassembly and assembly path planning, drawn according to points x, y, and z, is shown in Figure 9 [23].

Based on the principle of work (Equation (4)), the experimental results indicated that when the workbench height was set to 80 cm, 100 cm, and 120 cm, the total energy required for the disassembly task was 15,461.24 joules, 10,267.30 joules, and 23,015.46 joules, respectively. These findings suggest that a workbench height of 100 cm (aligned with the height of the machine) minimizes operator energy expenditure during the disassembly task. For operators of varying body heights, the conclusions presented in Section 4.1 of this study may serve as a reference for determining the most ergonomically suitable workbench height.

### 4.2. Experimental Result of the Smart Glove with FlexiForce Thin-Film Sensors

Using FlexiForce thin-film sensor gloves for disassembly/assembly work, the sensed data are analyzed in detail in this section. The experiment is simultaneously recorded to identify and confirm the subtle motions and force analysis during disassembly and assembly work.

- Force analysis of pneumatic piping disassembly/assembly

When disassembling or assembling the material sorting and processing machine, a total of twenty piping connectors, including six solenoid valves, four drilling cylinders (two upper and two lower), two stamping cylinders, and eight inductive anti-reverse valves, require disassembly and reassembly for the pneumatic piping. The piping connectors are divided into high connectors and low connectors based on their height, according to the operating surface of the dividing table, which is 135 mm in height. Piping connectors of the stamping cylinder and drilling cylinder (upper) being higher than the dividing table operating surface are regarded as high connectors, while piping connectors of the drilling cylinder (lower), check valve, and solenoid valve being lower than the dividing table operating surface are considered as low connectors.

**Figure 10:**
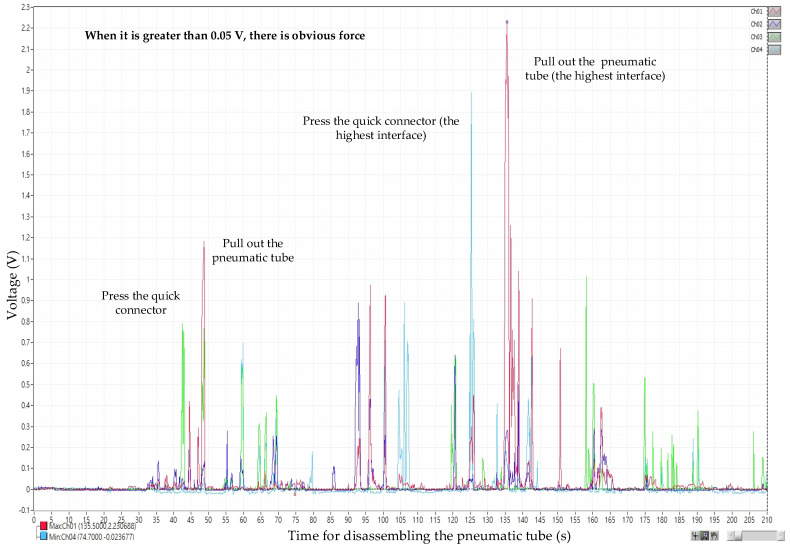
Voltage diagram for disassembling the high interface of the pneumatic tube.

Figure 10 shows the disassembly operation of the pneumatic tubing, where the left thumb and index finger (Ch03, Ch04) are used to press the “collet ring,” followed by the right thumb and index finger (Ch01, Ch02) performing the pulling action. In contrast, during the assembly of the pneumatic tubing, simply inserting the tube into the quick connector completes the connection. The detailed force application during these processes was analyzed. Analysis of the experimental data indicates that the average force applied during disassembly of the lower port section of the pneumatic tubing is 6.454 N, and during assembly, it is 6.185 N. For the upper port section, the average disassembly force is 7.639 N, while the assembly force is 7.072 N. Therefore, when assembling or disassembling the pneumatic tubing at the lower port, the applied forces are approximately 1.185 N and 0.887 N less, respectively, compared to those at the upper port.

- Disassembly/assembly screw force analysis

Screw disassembly/assembly force data with average force and better maximal force contain M2 slotted screws, M3 cross-head screws, M6 hex socket screws, and M8 hex socket screws, which are divided into fastening and loosening in the experiment.

**Figure 11:**
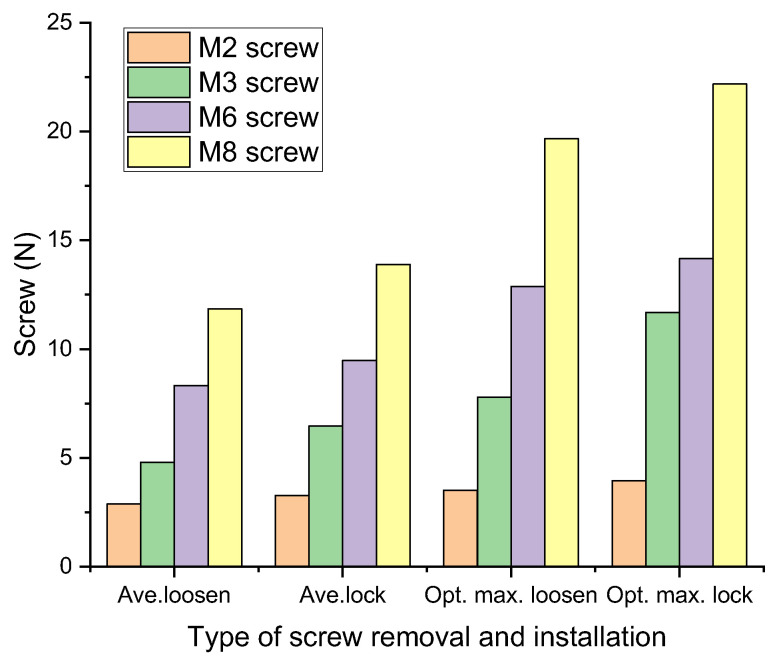
Comparison of average and optimal maximum force of screw removal and installation.

Figure 11 reveals that when the voltage is above 0.05 V, M2 and M3 screws precede disassembly and assembly work, while M6 and M8 screws precede disassembly and assembly work when the voltage is above 0.125 V. The maximum voltage for screw fastening is specified in ISO 898-7:1992 [24]. The minimal breaking torque for screw strength 8.8 is applied; the hex key used for disassembly/assembly work is fixed on a 7.5 mm lever arm of a wrench, and the thumb (Ch01), for screw disassembly/assembly work, is the major force finger.

Analysis of the experimental data shows that the average loosing force is 2.876 N, the average fastening force is 3.266 N, the better maximal loosing force is 3.517 N, and the better maximal fastening force is 3.955 N for the M2 screw; the average loosing force is 4.791 N, the average fastening force is 6.472 N, the better maximal loosing force is 7.79 N, and the better maximal fastening force is 11.678 N for the M3 screw; the average loosing force is 8.322 N, the average fastening force is 9.477 N, the better maximal loosing force is 12.878 N, and the better maximal fastening force is 14.157 N for the M6 screw; and the average loosing force is 11.853 N, the average fastening force is 13.883 N, the better maximal loosing force is 19.659 N, and the better maximal fastening force is 22.177 N for the M8 screw. In the comparison of screws, Figure 11 shows that the fastening force is larger. [25].

- Mechanism module disassembly/assembly analysis

Disassembly and assembly of the inductive anti-reverse valve, dividing table module, stamping cylinder module, and drilling cylinder module are preceded by the disassembly and assembly of the mechanism module. The adjustable table height was set at 80 cm, 100 cm, and 120 cm in the experiment to analyze the average force required for picking and placing modules on the adjustable work table during the disassembly and assembly processes based on the force differences at low, medium, and high adjustable table heights.

**Figure 12:**
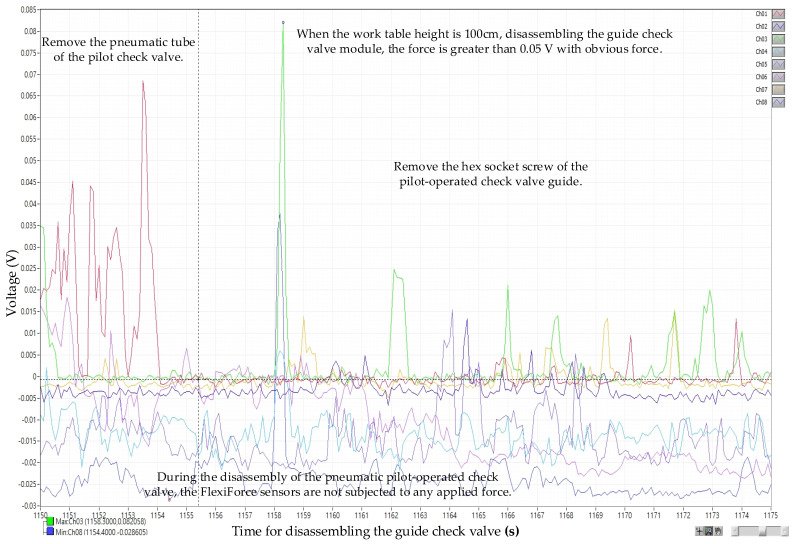
Voltage diagram for the disassembly of the guide check valve (work table height: 100 cm).

The selection range for effective voltage data is first set. The selection range for dividing table module, stamping cylinder module, and drilling cylinder module, except for the inductive anti-reverse valve, is between the minimal stamping cylinder 0.145 V and the maximal dividing table 2.135 V. Since the weight of inductive anti-reverse valve is relatively lighter than the rest three, the obvious force voltage 0.05 V, from the recording, is regarded as the minimal value, and the maximal value is the dividing table voltage 2.135 V, which is the same as the rest of the modules. The data analysis from disassembly picking/placing is shown in Figure 12, and the assembly picking/placing is shown in Figure 12, where the *X*-axis represents time (s) for recording voltage and the *Y*-axis represents the sensed voltage (V). By selecting the data in the disassembly/assembly timeline of the four major mechanism modules and exporting them into Excel files, the voltage of the fingers disassembling/assembling mechanism module (Ch01~Ch08) is averaged.

**Figure 13:**
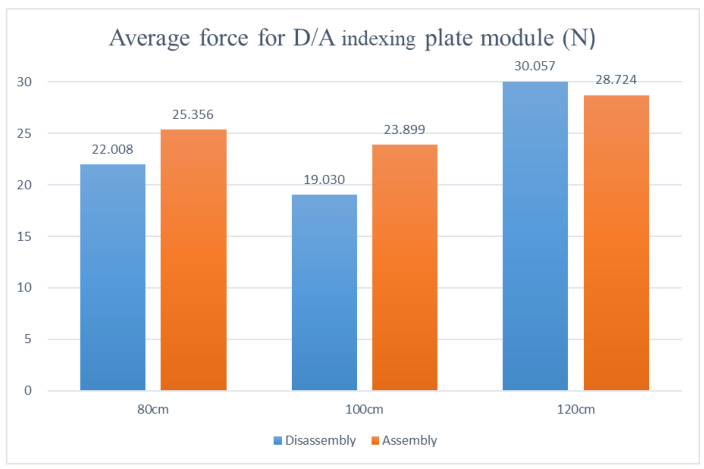
Force diagram for disassembling and assembling the index plate module at various work table heights.

**Figure 14:**
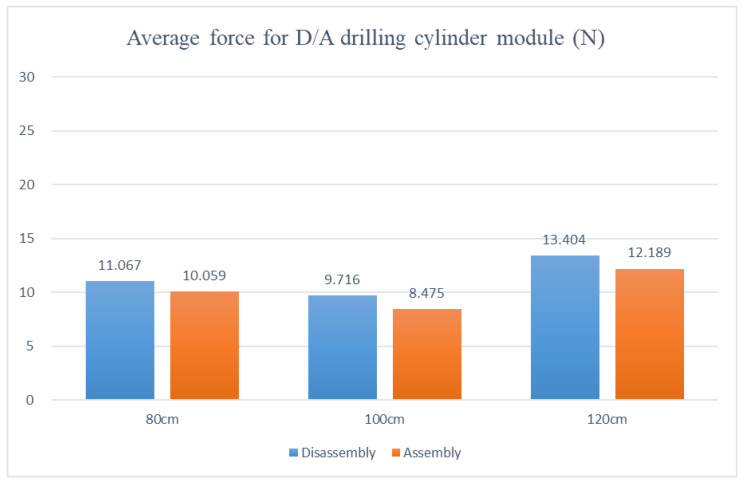
Force diagram for disassembling and assembling the drilling cylinder module at various work table heights.

**Figure 15:**
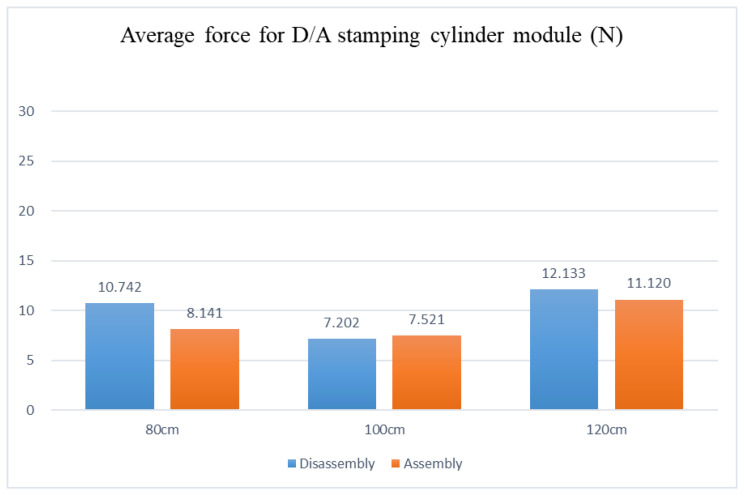
Force diagram for disassembling and assembling the stamping cylinder module at various work table heights.

**Figure 16:**
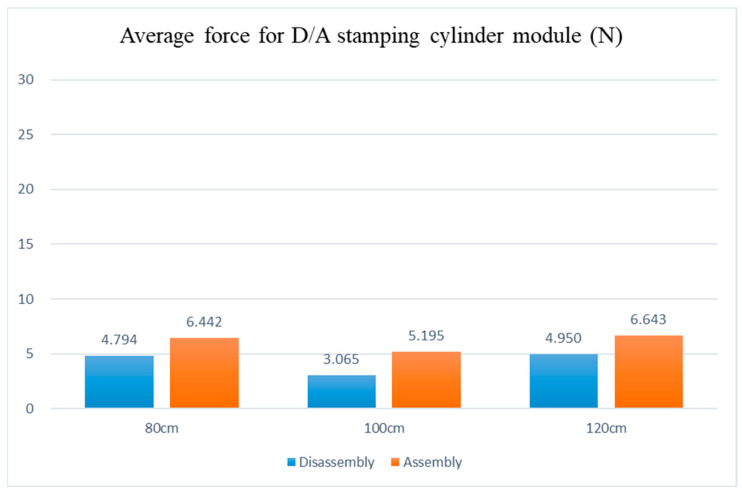
Force diagram for disassembling and assembling the guide check valve module at various work table heights.

Analysis of the experimental data shows that the disassembly and assembly average force, with the adjustable table at 80 cm, appears as the dividing table at 22.008 N and 25.356 N, the drilling cylinder at 11.067 N and 10.059 N, the stamping cylinder at 10.742 N and 8.141 N, and the inductive anti-reverse valve at 4.794 N and 6.442 N, respectively. The disassembly and assembly average forces, with the adjustable table at 100 cm, reveal the dividing table at 19.03 N and 23.899 N, the drilling cylinder at 9.716 N and 8.475 N, the stamping cylinder at 7.202 N and 7.521 N, and the inductive anti-reverse valve at 3.065 N and 5.195 N, respectively. The disassembly and assembly average forces, with the adjustable table at 120 cm, show the dividing table at 30.057 N and 28.724 N, the drilling cylinder at 13.404 N and 12.189 N, the stamping cylinder at 12.133 N and 11.120 N, and the inductive anti-reverse valve at 4.950 N and 6.643 N, respectively. After comparing the three, as shown in Figure 13, Figure 14, Figure 15 and Figure 16, the least force is observed on the adjustable table at 100 cm, while the largest force is observed on the adjustable table at 120 cm. In other words, when the adjustable desk height is set to 100 cm, the total applied force is the lowest at 84.103 N. Conversely, at a height of 120 cm, the total applied force reaches the maximum at 119.637 N. This indicates that an optimal workbench height can help the operator save between 14.3933% and 35.2579% of physical effort.

## 5. Conclusions

By having operators wear self-made smart gloves and inertial measurement sensors, the work tables with three different heights are equipped with a mechatronics machine disassembly and assembly experiment. The measured force, location, speed, and acceleration are applied to verify the functional best path for machine disassembly and assembly, as determined by the performance measure. Aiming at the experimental research on the application of inertial measurement units to mechatronics equipment disassembly/assembly and the performance index function value analysis in the disassembly/assembly process, the conclusions are explained as follows:

1. Placing the work table on the side of the operator’s dominant hand is the best placement location to reduce work energy consumption and enhance work efficiency.
2. Factory workers differ in their roles during the disassembly and assembly processes. The best work table height could be adjusted to be 5–10 cm higher than the elbow height of standing factory workers. The optimal work table height design equation for factory workers, based on the average standard height of Chinese adults (as specified in the Chinese standard GB10000-1988 [26]), is also provided.
3. The best disassembly and assembly sequence, the shortest time, and the optimal path planning for machine disassembly and assembly are also provided.
4. A wearable disassembly and assembly monitoring prototype system has been successfully developed, and a reference model for disassembly and assembly motion and force data has also been established.

Aiming to conduct experimental research on the application of self-made wearable smart gloves to machine disassembly and assembly, the subtle information of factory workers’ motions and the performance index function value analysis in the disassembly and assembly process are acquired as follows:

1. When disassembling and assembling pneumatic piping, working on connectors that are lower than the 135 mm dividing table shows a smaller force, while a larger force appears when working on connectors that are higher than the 135 mm dividing table. Regarding the situation of the machine in this experiment, the pneumatic connector could be designed to be close to 100 cm, as a better force height for disassembly and assembly work could reduce the force on the pneumatic interface.
2. Regarding common screws (M2, M3, M6, and M8) for material sorting and processing machines, the maximum loosening or fastening value and the average force for loosening or fastening are preferable. Additionally, a slightly larger force for fastening screws than for loosening screws is beneficial. For this reason, using average force and maximal loosening/fastening force for wiring screws or mechanism screw disassembly/assembly could enhance efficiency in the experiment and avoid broken screws caused by excessive applied force.
3. In the disassembly/assembly process of the dividing table module, drilling cylinder module, stamping cylinder module, and inductive anti-reverse valve module, the least force required for picking and placing the four mechanism modules is observed at an adjustable table height of 100 cm, i.e., when the parallel adjustable table height and machine height are optimized. Consequently, when preceding disassembly, assembly, or maintenance, modules can be placed on the surface parallel to the machine height for the mechanism module or parts picking and placing, effectively reducing the force applied.

The current research has limitations and areas for improvement. Equipment, process, and precision could be optimized or corrected in the future. The following research directions are suggested.

This study captures and analyzes the data from inertia sensors and thin-film sensors on smart gloves during the disassembly and assembly processes. To gain a deep understanding of motion gestures and behavior analysis at different states (e.g., carrying heavy objects), information acquired from other sensors should be taken into account.

The disassembly and assembly steps in the current research are preceded by manual labor. It is suggested to install the system on automation robots or robotic arms for the experiment to compare the data differences and further optimize the data and process. It is also expected that it could be applied to education and training certificate examination standards to enhance disassembly and assembly efficiency, reduce danger, and gradually optimize processes.

This study is expected to provide a reference for effectively identifying specific disassembly/assembly behaviors and predicting abnormal operating models. It also presents potential applications in more intricate disassembly and assembly process optimization, ergonomic analysis, worker training systems, operation fatigue and error warning systems, human–machine collaborative disassembly and assembly work systems, and intelligent robot disassembly and assembly work systems.

## Data Availability

The original contributions presented in this study are included in the article. Further inquiries can be directed to the corresponding author.

## Abbreviations

The following abbreviations are used in this manuscript:

D/A	Disassembly and Assembly
IMU	Inertial Measurement Unit
PMF	Performance Measure Functional

## References

[1] Mittal S., Khan M.A., Romero D., Wuest T. (2019). Smart manufacturing: Characteristics, technologies and enabling factors. Smart Manuf. Digit. Fact..

[2] Büchi G., Cugno M., Castagnoli R. (2020). Smart factory performance and Industry 4.0. Technol. Forecast. Soc. Change.

[3] Lettera G., Costa D., Callegari M. (2025). A Hybrid Architecture for Safe Human–Robot Industrial Tasks. Appl. Sci..

[4] Park Y., Hong J. (2025). Autoencoder-Based DIFAR Sonobuoy Signal Transmission and Reception Method Incorporating Residual Vector Quantization and Compensation Module: Validation Through Air Channel Modeling. Appl. Sci..

[5] Cintra Faria A.C., Barbalho S.C.M. (2023). Mechatronics: A Study on Its Scientific Constitution and Association with Innovative Products. Appl. Syst. Innov..

[6] Asif M.E., Rastegarpanah A., Stolkin R. (2024). Robotic disassembly for end-of-life products focusing on task and motion planning: A comprehensive survey. J. Manuf. Syst..

[7] Iacob R., Popescu D., Mitrouchev P. (2012). Assembly/Disassembly Analysis and Modeling Techniques: A Review. Stroj. Vestn. J. Mech. Eng..

[8] Schmaljohann F., Hagedorn D., Buß A., Kumme R., Löffler F. (2012). Thin-film sensors with small structure size on flat and curved surfaces. Meas. Sci. Technol..

[9] Song L., Xu B.B., Cheng Q., Wang X., Luo X., Chen X., Chen T., Huang Y. (2021). Instant interfacial self-assembly for homogeneous nanoparticle monolayer enabled conformal "lift-on" thin film technology. Sci. Adv..

[10] Zheng T., Xu A., Xu X., Liu M. (2023). Modeling and Compensation of Inertial Sensor Errors in Measurement Systems. Electronics.

[11] García-de-Villa S., Casillas-Pérez D., Jiménez-Martín A., García-Domínguez J.J. (2023). Inertial Sensors for Human Motion Analysis: A Comprehensive Review. IEEE Trans. Instrum. Meas..

[12] Siciliano B., Sciavicco L., Villani L., Oriolo G. (2009). Robotics: Modelling, Planning and Control.

[13] Bryson A.E., Ho Y.-C. (1975). Applied Optimal Control: Optimization, Estimation, and Control.

[14] Garriz C., Domingo R. (2022). Trajectory Optimization in Terms of Energy and Performance of an Industrial Robot in the Manufacturing Industry. Sensors.

[15] Jeong W., Lee C., Lee N., Hong S., Kang D., An D. (2025). Improving Sensor Adaptability and Functionality in Cartographer Simultaneous Localization and Mapping. Sensors.

[16] Manrique-Cordoba J., de la Casa-Lillo M.Á., Sabater-Navarro J.M. (2025). N-Dimensional Reduction Algorithm for Learning from Demonstration Path Planning. Sensors.

[17] Chern J.S. (1990). A Study of Collision-Free Techniques and Path Optimization of Two Robot Arms. Master’s Thesis.

[18] Movella Holdings Inc. (2023). Xsens Motion Capture. https://www.movella.com.

[19] Tekscan (2016). Force Sensors, Tekscan Inc.. https://www.tekscan.com.

[20] Tamantini C., Marra F., Di Tocco J., Di Modica S., Lanata A., Cordella F., Ferrarin M., Rizzo F., Stefanelli M., Papacchini M. (2025). SenseRisc: An instrumented smart shirt for risk prevention in the workplace. Wearable Technol..

[21] Di Rienzo F., Virdis A., Vallati C., Carbonaro N., Tognetti A. A sensorized glove for industrial safety based on Near-Field Communication. Proceedings of the IEEE International Conference on Smart Computing.

[22] Ghandi S., Masehian E. (2015). Review and Taxonomies of Assembly and Disassembly Path Planning Problems and Approaches. Comput. Aided Des..

[23] Chen C.S., Zhang H.W. Path planning for disassembly and assembly of mechatronic equipment using inertial measurement devices. Proceedings of the 21st Precision Machinery and Manufacturing Technology Symposium.

[24] (2020). Mechanical Properties of Fasteners.

[25] Chen C.S., Jiang B.C. Application of Thin Film Pressure Sensors in Disassembly and Assembly Analysis of Mechatronic Equipment. Proceedings of the 41st National Symposium of the Chinese Society of Mechanical Engineering.

[26] (1988). Human Dimension of Chinese Adults.

